# The Prognostic Impact of NK/NKT Cell Density in Periampullary Adenocarcinoma Differs by Morphological Type and Adjuvant Treatment

**DOI:** 10.1371/journal.pone.0156497

**Published:** 2016-06-08

**Authors:** Sebastian Lundgren, Carl Fredrik Warfvinge, Jacob Elebro, Margareta Heby, Björn Nodin, Agnieszka Krzyzanowska, Anders Bjartell, Karin Leandersson, Jakob Eberhard, Karin Jirström

**Affiliations:** 1 Department of Clinical Sciences Lund, Oncology and Pathology, Lund University, 221 85, Lund, Sweden; 2 Center for Molecular Pathology, Department of Translational Medicine, Lund University, Lund, Sweden; INRS, CANADA

## Abstract

**Background:**

Natural killer (NK) cells and NK T cells (NKT) are vital parts of tumour immunosurveillance. However, their impact on prognosis and chemotherapy response in periampullary adenocarcinoma, including pancreatic cancer, has not yet been described.

**Methods:**

Immune cell-specific expression of CD56, CD3, CD68 and CD1a was analysed by immunohistochemistry on tissue microarrays with tumours from 175 consecutive cases of periampullary adenocarcinoma, 110 of pancreatobiliary type (PB-type) and 65 of intestinal type (I-type) morphology. Kaplan-Meier and Cox regression analysis were applied to determine the impact of CD56+ NK/NKT cells on 5-year overall survival (OS).

**Results:**

High density of CD56+ NK/NKT cells correlated with low N-stage and lack of perineural, lymphatic vessel and peripancreatic fat invasion. High density of CD56+ NK/NKT cells was associated with prolonged OS in Kaplan-Meier analysis (p = 0.003), and in adjusted Cox regression analysis (HR = 0.49; 95% CI 0.29–0.86). The prognostic effect of high CD56+ NK/NKT cell infiltration was only evident in cases not receiving adjuvant chemotherapy in PB-type tumours (p for interaction = 0.014).

**Conclusion:**

This study demonstrates that abundant infiltration of CD56+ NK/NKT cells is associated with a prolonged survival in periampullary adenocarcinoma. However, the negative interaction with adjuvant treatment is noteworthy. NK cell enhancing strategies may prove to be successful in the management of these cancers.

## Background

Adenocarcinomas originating in the pancreas and the periampullary region are heterogeneous, with the shared common trait of being highly malignant and having a dismal prognosis. Despite the undisputable beneficial effect of chemotherapy [1, 2], pancreatic cancer is the fourth leading cause of cancer death, with a 5-year survival rate of 7%, although that might be an overestimate due to wrongly coded registers. If the disease presents at an advanced stage, the 5-year survival rate is only 2%, with a median survival of 5–8 months [2, 3]. Thus, there is an urgent need to identify new prognostic and treatment predictive markers so as to enable optimized individual therapy and improve outcomes for these patients.

Natural killer (NK) cells are vital in immunosurveillance and early clearance of tumour cells [4], as well as in preventing progression and spread of cancer [5]. This is effectuated by NK-cell-mediated cytotoxicity, antibody dependent cellular cytotoxicity (ADCC) as well as cytokine secretion [6], activated by lack of surface receptors on target cells such has MHC-I or by the presence of activating surface molecules [6]. Natural killer T cells (NKT) have a similar role in immunosurveillance as NK cells, but are activated primarily by CD1d recognition [7]. However, their role in anti-tumour response has not yet been mapped as extensively as for NK cells [7].

Previous research has shown a down-regulation of NK cell infiltration along with tumour progression in gastric, colorectal and oesophageal cancer [8, 9]. Furthermore, associations between NK cell infiltration and an improved survival have been observed in non-small cell lung cancer (NSCLC) and colorectal cancer [10, 11].

To the best of our knowledge, the occurrence and prognostic implications of NK/NKT-cells in periampullary adenocarcinoma have not yet been described. Therefore, the aim of this study was to analyse the clinicopathological correlates and prognostic significance of the density of CD56+ NK/NKT cells in the tumour microenvironment of periampullary adenocarcinoma, including pancreatic cancer, with particular reference to morphological type. In addition, the prognostic impact of tumour-cell specific CD56 expression was explored.

## Materials and Methods

### Study cohort

The study was approved of by the Ethics committee of Lund University (ref nr 445/07). whereby the committee waived no need for consent other than by the option to opt out. All patient data were anonymized and de-identified prior to analysis.

The study cohort is a retrospective consecutive series consisting of all primary tumours from 175 patients with periampullary adenocarcinoma, surgically treated with pancreaticoduodenectomy in the university hospitals of Malmö and Lund, Sweden, from January 1 2001 to December 31 2013. Information on vital status was obtained from the Swedish National Civil Register. Follow-up started at the date of surgical treatment and ended at the date of death, 5 years after surgery or December 31 2013. Data on adjuvant treatment were obtained from patient charts. All cases have been histopathologically re-evaluated, and 110 cases were classified as being of pancreatobiliary-type (PB-type) and 65 cases as being of intestinal-type (I-type) [12].

### Tissue microarray construction and immunohistochemistry

Tissue microarrays (TMA) were constructed as previously described [13, 14], using a semi-automated arraying device (TMArrayer, Pathology Devices, Westminister, MD, USA). A set of three 1 mm cores was obtained from viable areas of the 175 primary tumours. For immunohistochemical IHC analysis of CD56, CD3, CD68 and CD1A, 4 μm TMA-sections were pre-treated using ULTRA Cell Conditioning Solution 1, pH 8.5 (Ventana Medical Systems Inc., Tucson, AZ, USA) for heat induced epitope retrieval, and stained in a Ventana BenchMark stainer (Ventana Medical Systems Inc.) with the following antibodies: CD56: Clone MRQ-42, pre-diluted, Ventana Medical Systems Inc., CD3: Clone 2GV6, pre-diluted, Ventana Medical Systems Inc., CD1a: clone NCL-CD1a-220, diluted 1:25, LEICA Biosystems, Newcastle, UK, CD68: clone KP1, diluted 1:1000, Dako, Glostrup, Denmark. The antibody-antigen complex was visualized with ultraView Universal DAB Detection kit (Ventana Medical Systems Inc.).

### Immunohistochemical staining assessment

The total number of CD56+ lymphocytes was counted in each TMA core, and their localisation within the tissue landscape was denoted, either as intratumoural (defined as tumour cell and CD56+ lymphocyte being juxtaposed), within the adjacent stroma (defined as CD56+ lymphocyte being within one tumour cell diameter from a tumour cell) or within the distant stroma (defined as being more than one tumour cell diameter from a tumour cell) [15]. The total number of CD3+ lymphocytes, CD1a+ immune cells and CD68+ immune cells was counted in each core and a median was used in subsequent analyses. Manual counting for CD3 was verified by automated analysis using the Object Colocalization algorithm within the Halo image analysis software (Indica Labs, Corrales, NM, USA). Tumour-specific expression of CD56 was denoted as the fraction of positive cells and staining intensity. A median of all core values for each case was used in the subsequent statistical analyses. In order to create an NK/NKT cell/tissue ratio, the proportion of tumour, stroma and “other” (benign-appearing tissue or missing parts) was estimated in all cores.

### Statistics

Mann-Whitney U-test was applied to assess differences in distribution of the investigative factors in relation to clinicopathological characteristics. Paired T-test was used to demonstrate any associations between different types of immune cell infiltrates. Classification and regression tree (CRT) analysis was applied in order to find an optimal prognostic cut-off. Two patients with PB-type adenocarcinomas who received neoadjuvant therapy were excluded from the statistical analyses. In addition, three patients were excluded from the survival analyses; one with PB-type adenocarcinoma who emigrated and two with I-type adenocarcinomas who died within one month of surgical treatment due to complications.

Kaplan-Meier analysis and log rank test were applied to illustrate any difference in 5-year overall survival (OS) and recurrence free survival (RFS). Cox regression proportional hazard models were used to estimate hazard ratios (HR) in both uni- and multivariable analysis, adjusted for age, T-stage, N-stage, differentiation grade, lymphatic and vascular infiltration, perineural growth and adjuvant chemotherapy. In order to create an NK/NKT cell/tissue ratio, a variable was constructed as follows: (Compartment specific CD56+ NK/NKT cell count/fraction of respective tissue) and a sum of all three compartment ratios was used in the subsequent analysis. To estimate the interaction of NK/NKT cell density with adjuvant chemotherapy, a variable was constructed of adjuvant treatment (high/low) x CD56+ (high/low). All calculations were performed with SPSS version 22.0 (SPSS Inc, Chicago, IL, USA). All statistical tests were two-sided and p-values < 0.05 were considered significant.

## Results

### Associations of immune cell-specific and tumour-specific CD56 expression with clinicopathological characteristics

**Fig 1:**
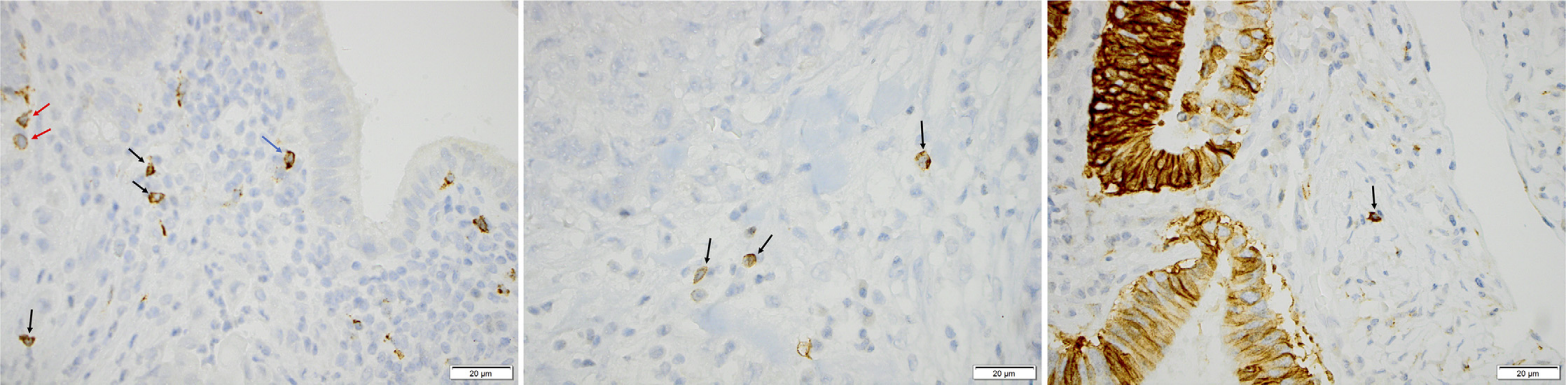
Sample immunohistochemical images. Immunohistochemical images of CD56 staining in periampullary adenocarcinoma. Sample images (40X magnification) visualising the presence of intra-tumoural (red arrow), stromal (black arrow) and tumour-adjacent (blue arrow) CD56+ NK/NKT cells in three different cases with A) papilla-ampulla intestinal origin, total CD56+ NK/NKT cell count 10, and tumour-specific CD56 expression 0, B) distal bile duct origin, total CD56+ NK/NKT cell count 6 and tumour-specific CD56 expression C) pancreas origin, total CD56+ NK/NKT cell count 5, and tumour-specific CD56 expression 100%.

Immune cell-specific and tumour-specific CD56 expression was assessable in 168 (96.0%) cases, of which 62 (95.38%) were I-type and 106 (96.36%) PB-type. Tumour-specific CD56 expression was assessable in 156 (89.14%) cases, of which 61 (93.84%) were I-type and 95 (86.36%) PB-type. Sample immunohistochemical images are shown in Fig 1.

Associations between CD56+ NK/NKT-cell density and established clinicopathological parameters by morphological type are shown in Table 1. In PB-type tumours, CD56+ NK/NKT-cell density was borderline significantly associated with absence of lymphatic growth (p = 0.050). In I-type tumours, CD56+ NK/NKT-cell density was inversely associated with N-stage (p = 0.004), perineural growth (p = 0.012), lymphatic growth (p = 0.004) and peripancreatic fat infiltration (p = 0.001).

**Table 1:** Associations between CD56+ NK/NKT cell count and clinicopathological factors.

	Pancreatobiliary-type		Intestinal-type	
Factor (n = pancreatobiliary-type, n = intestinal type)	Total CD56 median (range)	p-value	Total CD56 median (range)	p-value
Age*		0.742		0.706
Q1 (n = 19; n = 18)	1.00 (0.00–63.00)		2.00 (0.00–6.00)	
Q2 (n = 30; n = 13)	1.00 (0.00–8.50)		1.00 (0.00–9.00)	
Q3 (n = 25; n = 18)	1.00 (0.00–10.00)		1.50 (0.00–22.50)	
Q4 (n = 29; n = 11)	1.00 (0.00–11.50)		1.00 (0.00–5.00)	
Sex		0.781		0.606
Female (n = 48; n = 34)	1.00 (0.00–12.00)		1.75 (0.00–9.00)	
Male (n = 56; n = 34)	0.75 (0.00–63.00)		1.00 (0.00–22.50)	
Differentiation grade		0.306		0.172
Poor (n = 38; n = 30)	1.00 (0.00–63.00)		1.50 (0.00–22.50)	
Well and moderate (n = 66; n = 32)	0.75 (0.00–12.00)		1.00 (0.00–5.50)	
Tumour stage		0.091		0.186
T1 and T2 (n = 12; n = 14)	2.25 (0.00–10.00)		2.00 (0.00–4.50)	
T3 and T4 (n = 92; n = 48)	1.00 (0.00–63.00)		2.00 (0.00–4.50)	
Nodal stage		0.269		**0.004**
N0 (n = 30; n = 32)	1.00 (0.00–63.00)		2.25 (0.00–22.50)	
N1 (n = 43; n = 19)	0.50 (0.00–8.50)		0.00 (0.00–3.00)	
N2 (n = 31; n = 11)	1.00 (0.00–12.00)		0.00 (0.00–6.00)	
Resection margins		0.741		0.406
R0 (n = 6; n = 17)	0.00 (0.00–8.00)		1.50 (0.00–22.50)	
R1 (n = 7; n = 14)	1.00 (0.00–63.00)		0.00 (0.00–6.00)	
RX (n = 20; n = 31)	0.50 (0.00–12.00)		2.00 (0.00–9.00)	
Perineural growth		0.340		**0.012**
Absent (n = 22; n = 43)	1.00 (0.00–12.00)		2.00 (0.00–22.50)	
Present (n = 82; n = 19)	1.00 (0.00–63.00)		0.00 (0.00–6.00)	
Lymphatic growth		0.050		**0.004**
Absent (n = 31; n = 29)	2.00 (0.00–63.00)		2.00 (0.00–22.50)	
Present (n = 73; n = 33)	0.50 (0.00–12.00)		1.00 (0.00–5.50)	
Vascular growth		0.560		0.363
Absent (n = 67; n = 57)	1.00 (0.00–63.00)		1.50 (0.00–22.50)	
Present (n = 37; n = 5)	1.00 (0.00–11.50)		0.00 (0.00–5.50)	
Peripancreatic fat growth		0.641		**0.001**
Absent (n = 22; n = 40)	0.75 (0.00–63.00)		2.00 (0.00–22.50)	
Present (n = 82; n = 22)	1.00 (0.00–12.00)		0.00 (0.00–6.00)	

* Q1 = 38–61, Q2 = 62–67, Q3 = 68–72, Q4 = 73–84

Paired T-test revealed significant correlations between CD56+ NK/NKT-cell infiltration and CD3+ lymphocyte infiltration (p <0.001), CD1a+ DC infiltration (p = 0.005) and CD68+ myeloid cell infiltration (p = 0.030), respectively.

Associations of tumour-specific CD56 expression with clinicopathological factors are shown in S1 Table. Notably, tumour-specific CD56+ expression was higher in cases with peripancreatic tumour growth (p = 0.006) and involved margins (p = 0.046).

### Prognostic significance of immune cell-specific and tumour-specific CD56 expression

**Fig 2:**
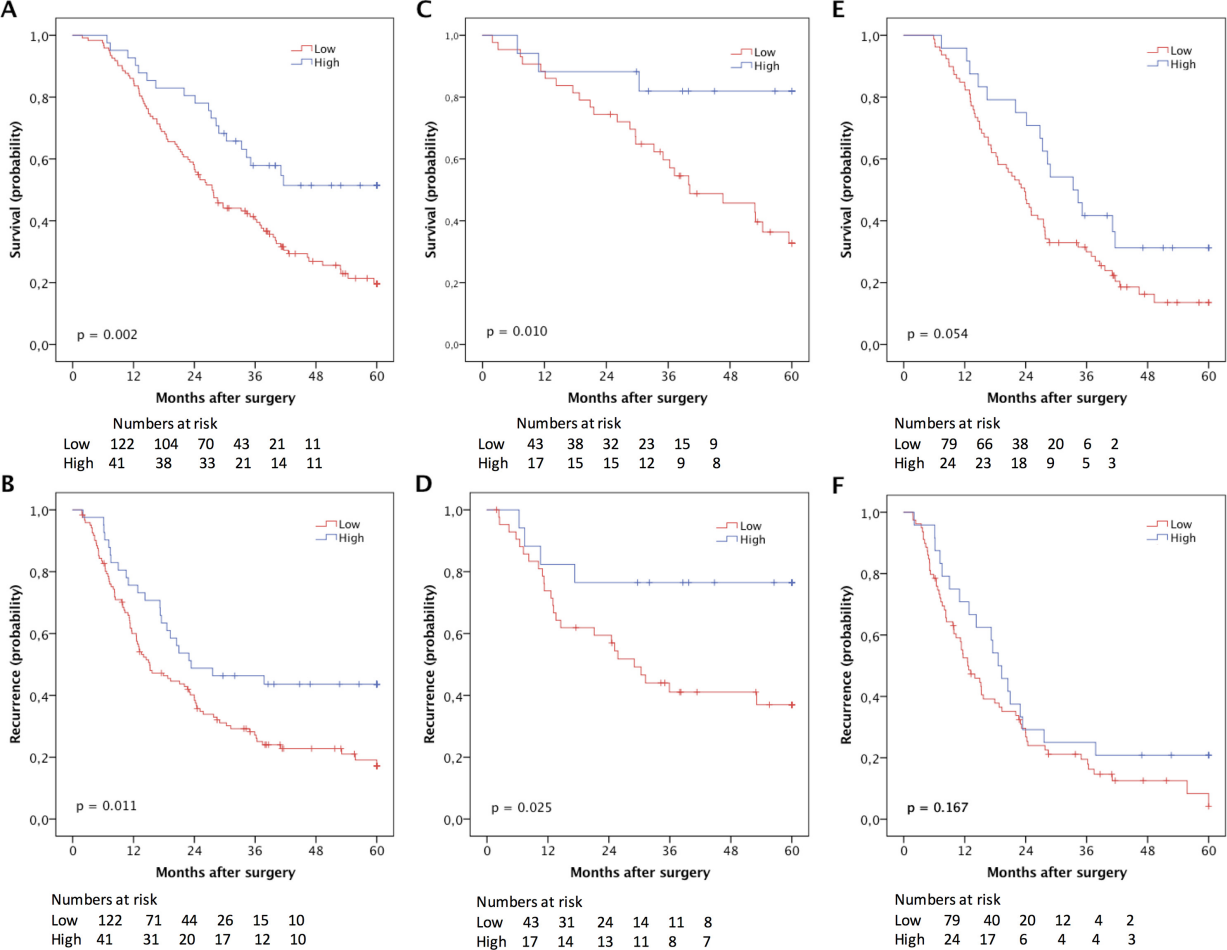
Kaplan-Meier estimates of survival according to NK/NKT cell density. Kaplan-Meier estimates of 5-year overall survival according to high and low CD56+ NK/NKT cell count in (A) the entire cohort, (C) in I-type tumours and (E) in PB-type, and recurrence free survival in (B) the entire cohort, (D) in I-type tumours and (F) in PB-type tumours.

CRT analysis established an optimal prognostic cut-off at 2.75 for the total CD56+ NK/NKT cell count, whereby 41 cases were defined as having high and 125 cases as having low NK/NKT cell density. Kaplan-Meier analysis of the entire cohort demonstrated a significantly prolonged OS for patients with high CD56+ NK cell density (p = 0.002) (Fig 2). Kaplan-Meier analysis in strata according to morphological type demonstrated a significant association between high NK/NKT cell density and prolonged OS in I-type tumours (p = 0.010) but borderline significant in PB-type tumours (p = 0.054) (Fig 2). This association was confirmed in univariable Cox regression analysis for I-type tumours (HR = 0.23; 95% CI 0.71–0.78) and in PB-type tumours (HR = 0.59; 95% CI 0.34–1.02) (Table 2). In multivariable analysis, the significance was lost for I-type tumours but nearly reached significance in PB-type tumours (HR = 0.59; 95% CI 0.33–1.06) (Table 2).

**Table 2:** Cox proportional hazards analysis of the impact of CD56+ NK/NKT cell infiltration on overall survival according to adjuvant treatment in intestinal-type and pancreatobiliary-type adenocarcinomas.

CD56+ NK/NKTcell count			P for interaction
Intestinal-type tumours			
*All*	*unadjusted*		
Low	1.00	43 (26)	
High	**0.23 (0.07–0.78)**	17 (3)	
	*adjusted*		
Low	1.00	43 (26)	
High	0.47 (0.11–1.95)	17 (3)	
*No adjuvant treatment*	*unadjusted*		*0*.*515*
Low	1.00	30 (21)	
High	**0.19 (0.04–0.80)**	12 (2)	
	*adjusted*		
Low	1.00	30 (21)	
High	0.46 (0.09–2.27)	12 (2)	
*Any adjuvant treatment*	*unadjusted *		
Low	1.00	13 (5)	
High	0.46 (0.05–3.93)	5 (1)	
	*adjusted*		
Low	1.00	13 (5)	
High	0.00 (0.00–1.94)	5 (1)	
Pancreatobiliary-type tumours			
*All*	*unadjusted*		
Low	1.00	79 (64)	
High	0.59 (0.34–1.02)	24 (16)	
	*adjusted*		
Low	1.00	79 (64)	
High	0.59 (0.33–1.06)	24 (16)	
*No adjuvant treatment*	*unadjusted *		***0*.*023***
Low	1.00	39 (34)	
High	**0.27 (0.09–0.76)**	9 (4)	
	*adjusted*		
Low	1.00	39 (34)	
High	**0.17 (0.05–0.55)**	9 (4)	
*Any adjuvant treatment*	*unadjusted *		
Low	1.00	40 (30)	
High	1.06 (0.54–2.09)	15 (12)	
	*adjusted*		
Low	1.00	40 (30)	
High	1.14 (0.54–2.42)	15 (12)	

Bold HRs indicate significant values. Adjusted analysis included age (continuous), T-stage (1–2 vs 3–4), N-stage, differentiation grade (poor vs well-moderate), lymphatic invasion, vascular invasion, perineural growth and adjuvant therapy (yes/no).

Kaplan-Meier analysis further demonstrated a significant association between high density of CD56+ NK/NKT cells and a prolonged RFS in the entire cohort (p = 0.011) and I-type tumours (p = 0.025), but not in PB-type tumours (Fig 2). Cox regression analysis confirmed the association between high CD56+ NK/NKT cell density and a reduced risk of recurrence in I-type tumours in univariable analysis (HR = 0.32; 95% CI 0.11–0.92), but this did not remain significant after adjustment for other prognostic factors (data not shown).

The prognostic impact of tissue compartment-specific CD56+ NK/NKT cell density, NK/NKT cell/tissue ratio and tumour-specific CD56 expression, respectively, in relation to OS is shown in in S1 Fig, S2 Fig, S3 Fig, S4 Fig, S1 Table and S2 Table. The count of tumour-adjacent CD56+ NK/NKT cells was too low to allow for any meaningful statistical analysis. Tumour-specific expression of CD56 was found to be an independent factor of poor prognosis in the whole cohort and in I-type tumours (S2 Table).

### Prognostic impact of CD3+ lymphocyte infiltration, CD56+/CD3+ lymphocyte ratio and CD56+/ CD1a+ and CD68+ leukocyte ratio

CRT analysis established an optimal prognostic cut off for CD3+ lymphocyte infiltration at 142.5, whereby 82 cases were classified as having low infiltration and 89 as high infiltration. The prognostic impact of CD3+ lymphocyte infiltration as illustrated by Kaplan-Meier analysis showed a significantly improved prognosis for patients with high CD3+ lymphocyte infiltration in I-type (p = 0.005) and PB-type (p = 0.006) tumours (S5 Fig). Inter-observer variability analysis revealed a substantial agreement between automated and manual analysis of CD3+ lymphocyte infiltration (κ = 0.733; p < 0.001).

Kaplan-Meier analysis of automated CD3+ infiltration revealed a significantly prolonged OS for patients with high CD3+ lymphocyte infiltration in the whole cohort (p = 0.001), in I-type tumours (p = 0.021), but not in PB-type tumours (S5 Fig). Univariable Cox regression analysis of CD3+ lymphocyte count showed a significantly improved OS for patients with high density in the whole cohort (HR = 0.42; 95% CI 0.29–0.62; p <0.001), in I-type tumours (HR = 0.37; 95% CI 0.18–0.76; p = 0.007), and in PB-type tumours (HR = 0.54; 95% CI 0.34–0.84; p = 0.007). The association remained significant in multivariable analysis in the whole cohort (HR = 0.52; 95% CI 0.35–0.78; p = 0.002) and in PB-type tumours (HR = 0.51; 95% CI 0.31–0.84; p = 0.008), but not in I-type tumours (HR = 0.43; 95% CI 0.17–1.04; p = 0.062).

Univariable Cox regression analysis of automated CD3+ lymphocyte count showed a significantly prolonged OS for patients with high density in the whole cohort (HR = 0.51; 95% CI 0.34–0.75; p = 0.001), in I-type tumours (HR = 0.44; 95% CI 0.21–0.90; p = 0.021) but not in PB-type tumours (data not shown). The association remained significant in multivariable analysis the whole cohort (HR = 0.66; 95% CI 0.44–0.99; p = 0.046) but not in strata according to morphological type.

A CD56+ lymphocyte/CD3+ lymphocyte ratio was created (cut off established using CRT analysis at 0.007), whereby 89 cases were classified as having a low ratio and 77 as having a high ratio. Kaplan-Meier analysis revealed significantly improved prognosis for patients with a high ratio in the whole cohort (p = 0.011) and I-type (p = 0.011), but not in PB-type tumours (S6 Fig). The association was significant in univariable Cox regression analysis in the whole cohort (HR = 0.61; 95% CI 0.41–0.89; p = 0.011) and in I-type tumours (HR = 0.37; 95% CI 0.17–0.83; p = 0.015), but did not remain significant in multivariable analysis, neither in the whole cohort nor in I-type tumours (data not shown).

Next, a CD56+ NK/NKT cell/CD1a+ and CD68+ immune cell ratio was created (cut off established at 0.017 using CRT analysis), whereby 101 cases were classified as having a low ratio and 61 as having a high ratio. Kaplan-Meier analysis revealed a significantly improved prognosis for patients with a high ratio in the whole cohort (p < 0.001), in PB-type (p = 0.022) but not in I-type tumours (p = 0.079) (S7 Fig). The association was significant in univariable Cox regression analysis of the whole cohort (HR = 0.46; 95% CI 0.32–0.72; p < 0.001), and in PB-type tumours (HR = 0.57; 95% CI 0.35–0.93; p = 0.024) but did not remain significant in multivariable analysis, neither in the whole cohort nor in PB-type tumours (data not shown).

### Prognostic impact of CD56+ lymphocytes in relation to adjuvant chemotherapy

**Fig 3:**
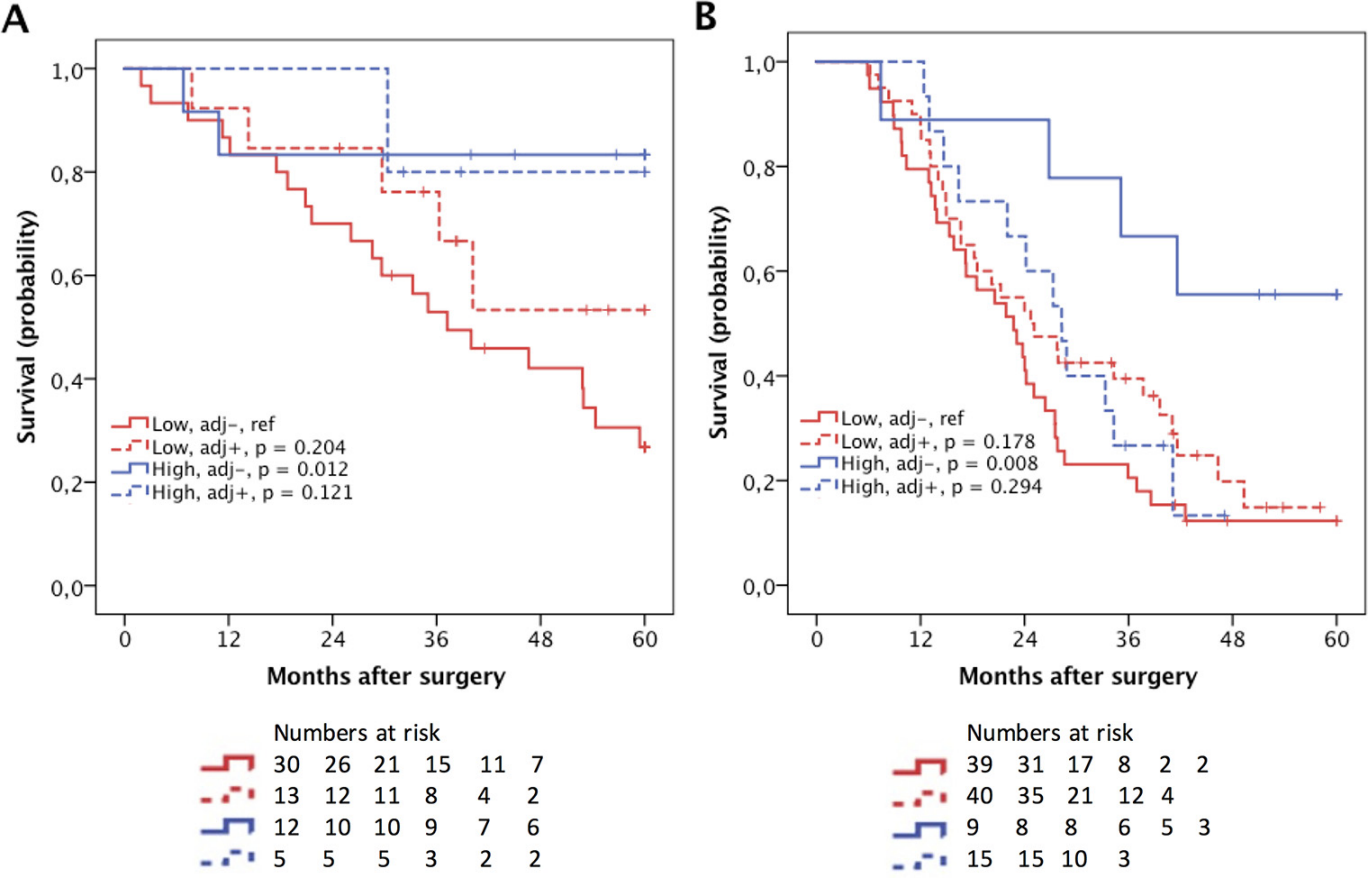
Kaplan-Meier estimates of survival according to NK/NKT cell density and adjuvant chemotherapy. Kaplan–Meier estimates of 5-year overall survival in combined strata according to high and low CD56+ count and adjuvant chemotherapy, respectively, in (A) I-type tumours and (B) PB-type tumours.

The prognostic impact of CD56+ NK/NKT cells in strata according to adjuvant chemotherapy and morphological type is shown in Fig 3. Patients with PB-type tumours with high CD56+ NK/NKT-cell density who had not received adjuvant therapy had a significantly prolonged OS (p = 0.008) compared to the reference group (low NK/NKT cell count, not receiving adjuvant therapy) as did patients with I-type tumours (p = 0.012) (Fig 3). A significant interaction between high density of CD56+ NK/NKT cells and adjuvant treatment was observed in PB-type tumours (p = 0.023) but not in I-type tumours (Table 2).

The prognostic impact of CD56+ NK/NKT cell infiltration compensated for the sum of CD1a+ and CD68+ leukocyte infiltration in strata according to adjuvant chemotherapy is shown in S8 Fig. In the whole cohort, patients who had a high NK/NKT cell ratio and who did not receive any adjuvant chemotherapy had a significantly prolonged OS (p <0.001). The same association was seen in patients with I-type tumours (p = 0.044). In patients with PB-type tumours, the trend was similar, but did not reach significance (p = 0.002). A significant treatment interaction between a high ratio of CD56+ NK/NKT cells in relation to the sum of CD68+ and CD1a+ cells were seen in the whole cohort (p = 0.007) and in PB-type tumours (p = 0.012), but not in I-type tumours (data not shown).

## Discussion

This study provides a first demonstration of the clinicopathological correlates and prognostic significance of CD56+ lymphocyte infiltration in periampullary adenocarcinoma, including pancreatic cancer. The results demonstrate that high density of NK/NKT cell infiltration is associated with an improved survival, in particular in I-type tumours.

These findings are in accordance with findings from studies on other types of gastrointestinal cancer such as oesophageal, gastric and colorectal cancer [8, 9, 11], as well as in NSCLC [10], linking NK cell density to a more favourable prognosis. Due to the low survival rates for patients with periampullary adenocarcinoma, even with aggressive treatment regimens, there is an ongoing pursuit to identify new therapeutic targets and strategies. NK cell function can be enhanced by several means, e.g. autologous cell transfer, cytokine therapy and monoclonal antibodies [16]. NK cell therapy has shown clinical promise in haematological malignancies, although some therapies merely increase the peripheral NK cell count, but not the tissue resident count [17]. Moreover, studies have also shown promise of NK cell based therapy in solid tumours; In NSCLC, adjuvant autologous cellular therapy and treatment with cytokine induced NK cells were both found to improve survival in combination with chemotherapy, compared to chemotherapy alone [18], and in gastric adenocarcinoma, adjuvant cellular immunotherapy in combination with chemotherapy was found to improve progression-free survival compared to adjuvant chemotherapy alone, however, there was no difference in OS after 2 years [19].

Another potentially important finding in the present study was that high CD56+ lymphocyte density was only prognostic in patients not receiving adjuvant chemotherapy, whereas in patients receiving adjuvant chemotherapy, CD56+ NK/NKT cell density had no impact on prognosis. This observation was particularly evident in PB-type tumours, where a significant interaction between adjuvant treatment and CD56+ NK/NKT cell density was found. Indeed, studies on breast cancer have demonstrated a negative impact of adjuvant chemotherapy on both the circulating pool of NK cells as well as on their function [20, 21]. In light of these findings, it can be hypothesised that patients with a high density of NK cells in their tumours will not benefit from standard chemotherapy, since this will negatively affect the tumour-inhibiting functions of the NK cell population. Of note, the herein investigated cohort is clinically well characterised, and only approximately half of the patients have been given adjuvant chemotherapy, which renders it useful for the identification of prognostic as well as potentially predictive biomarkers despite the retrospective setting. There are however limitations in that the number of cases available for subgroup analysis is small, e.g. when stratifying both for biomarker expression and adjuvant treatment. Nevertheless, as patients with periampullary cancer, in particular of PB-type, have a dismal prognosis, it is not only important to identify novel treatment strategies, but also to avoid unnecessary treatment leading to an impaired quality of life due to side effects. Therefore, while different strategies to enhance NK cell responses to these tumours may prove to be effective, further research addressing the effects of standard adjuvant chemotherapy on the tissue resident NK cell population is warranted.

There is no optimal surface marker for NK cells, which poses a difficulty in studies such as this one. NK cells can be identified by expression of CD56, however CD56 may also be expressed on other lymphocyte populations, although to a lesser degree, and by some myeloid derived cell populations [22–24]. In the present study, we tried to mitigate the potential overlap of NK cell markers by putting CD56+ lymphocyte infiltration in relation to both CD3+ lymphocyte infiltration and myeloid cell infiltration. This revealed that a high ratio of CD56+ NK/NKT density in relation to the sum of CD1a+ and CD68+ myeloid cell infiltration was associated with a favourable prognosis. Furthermore, in the whole cohort, patients with tumours displaying a high CD56+ NK/NK T cell ratio who received no adjuvant chemotherapy had a more favourable prognosis than patients with a low CD56+ NK/NK T cell ratio who received adjuvant chemotherapy. This highlights the potential treatment-modulatory properties of CD56+ NK/NKT cells, that was seen also without compensation for myeloid cell infiltration. In addition to this observation, this study demonstrated that high CD56+ lymphocyte/CD3+ lymphocyte infiltration was associated with a favourable prognosis, putting further emphasis on the positive prognostic impact of CD56+ lymphocyte infiltration. Since NKT cells express CD3 this would also suggest that NK cells alone probably have a positive prognostic impact.

There is evidence of synergistic cross-talk between NK cells and a wide spectrum of cell populations in the tumour microenvironment [6, 25, 26]. NK cell cytotoxicity and function is enhanced by chemokines secreted by DC and CD4+ T-cells such as IL-2, IL-12 and IL-15 [27]. However, T-reg lymphocytes, type 2 macrophages and tumour cells themselves can, by chemokine secretion, suppress NK cell activity and thus promote tumour escape [16, 27]. Future research is therefore warranted to further elucidate the relationship of NK/NKT cells with other components of the immune system with regard to chemotherapy efficacy, and to put NK cell function in a deeper context of regulatory immune cells in the tumour microenvironment.

In addition, this study found an adverse relationship between tumour-specific CD56+ expression and poor prognosis in periampullary adenocarcinoma, which is in line with previous findings [28]. Our study is however the first to show an independent prognostic value of tumour-specific CD56 expression in these cancers.

## Conclusion

This study provides a first demonstration of the prognostic impact of CD56+ lymphocytes in periampullary adenocarcinoma. While a high density of CD56+ NK/NKT cells was significantly associated with a prolonged survival, a negative interaction with adjuvant treatment was observed in patients with PB-type tumours. These findings indicate that NK/NKT cell enhancing strategies may prove to be successful in the management of these cancers, but also highlight the need for further studies addressing the potential negative modulatory effects of standard chemotherapy on NK/NKT cells.

## Supporting Information

S1 Fig Kaplan-Meier estimates of survival according to intra-tumour NK/NKT cell density. Kaplan-Meier estimates of 5-year survival according to intra-tumoural CD56+ NK/NKT cell count in (A) the entire cohort, (C) in I-type tumours and (E) in PB-type tumours, and recurrence free survival in (B) the entire cohort, (D) in I-type tumours, and (F) in PB-type tumours. CRT-analysis established a cut off of high (>0.75, n = 19) and low (≤0.75, n = 147) infiltration.
(DOCX)

S2 Fig Kaplan-Meier estimates of survival according to stromal NK/NKT cell density. Kaplan-Meier estimates of 5-year survival according to stromal CD56+ NK/NKT cell count in A) the entire cohort, (C) in I-type tumours and (E) in PB-type tumours, and recurrence free survival in (B) the entire cohort, (D) in I-type tumours, and (F) in PB-type tumours. CRT-analysis established a cut off of high (1.25, n = 56) and low (≤1.25, n = 115).
(DOCX)

S3 Fig Kaplan-Meier estimates of survival according to NK/NKT cell /tissue ratio. Kaplan-Meier estimates of 5-year survival according to CD56+ NK/NKT cell/tissue ratio in A) the entire cohort, (C) in I-type tumours and(E) in PB-type tumours, and recurrence free survival in (B) the entire cohort, (D) in I-type tumours, and (F) in PB-type tumours. CRT-analysis established a cut off of high (> 4.722, n = 133) and low (≤4.722, n = 22) infiltration.
(DOCX)

S4 Fig Kaplan-Meier estimates of survival according to tumour-specific CD56 expression. Kaplan-Meier estimates of 5-year survival according to tumour-specific CD56 expression in A) the entire cohort, (C) in I-type tumours and(E) in PB-type tumours, and recurrence free survival in (B) the entire cohort, (D) in I-type tumours, and (F) in PB-type tumours).
(DOCX)

S5 Fig Kaplan-Meier estimates of survival according to CD3+ lymphocyte infiltration. Kaplan-Meier estimates of 5-year survival according to CD3+ lymphocyte infiltration in A) the entire cohort, (B) in I-type tumours, (C) in PB-type tumours, (D) automated analysis of CD3+ infiltration in whole cohort, (E) automated analysis of CD3+ infiltration in I-type and (F) automated analysis of CD3+ infiltration in PB-type.
(TIFF)

S6 Fig Kaplan-Meier estimates of survival according to CD56+ lymphocyte to CD3+ lymphocyte infiltration ratio. Kaplan-Meier estimates of 5-year survival according to CD56+ lymphocyte to CD3+ lymphocyte infiltration ratio in A) the entire cohort, (B) in I-type tumours and (C) in PB-type tumours.
(TIFF)

S7 Fig Kaplan-Meier estimates of survival according to CD56+ lymphocyte to CD1a+ and CD68+ immune cell infiltration ratio. Kaplan-Meier estimates of 5-year survival according to CD56+ lymphocyte to CD1a+ and CD68+ immune cell infiltration in A) the entire cohort, (B) in I-type tumours and (C) in PB-type tumours.
(TIFF)

S8 Fig Kaplan-Meier estimates of survival according to CD56+ lymphocyte to CD1a+ and CD68+ immune cell infiltration ratio in reference to adjuvant chemotherapy. Kaplan–Meier estimates of 5-year survival in combined strata according to CD56+ lymphocyte to CD1a+ and CD68+ immune cell infiltration ratio and adjuvant chemotherapy in (A) the whole cohort, (B) in I-type tumours and (C) in PB-type tumours.
(TIFF)

S1 Table Associations between CD56+ tumour tissue fraction and clinicopathological factors. (DOCX)

S2 Table Cox proportional hazards analysis of the impact of intra-tumoural CD56+ NK/NKT cells, stromal NK/NKT cells, NK/NKT cell/tissue ratio and tumour-specific CD56 expression, respectively, on overall survival and recurrence-free survival. (DOCX)

## Abbreviations

ADCC: antibody dependent cellular cytotoxicity
CI: confidence interval
CRT: classification and regression tree
DC: dendritic cells
HR: hazard ratio
I-type: intestinal type
NK cell: Natural killer cell
NKT: Natural killer T cell
NSCLC: non-small cell lung cancer
OS: 5-year overall survival
PB-type: pancreatobiliary-type
RFS: recurrence free survival
