# Supplementary material for: The Prognostic Impact of NK/NKT Cell Density in Periampullary Adenocarcinoma Differs by Morphological Type and Adjuvant Treatment
Source: PLoS One. 2016 Jun 8;11(6):e0156497. doi: 10.1371/journal.pone.0156497 (PMC4898776; doi:10.1371/journal.pone.0156497)
Supplement: S1 Fig — Kaplan-Meier estimates of 5-year survival according to intra-tumoural CD56+ NK/NKT cell count in (A) the entire cohort, (C) in I-type tumours and (E) in PB-type tumours, and recurrence free survival in (B) the entire cohort, (D) in I-type tumours, and (F) in PB-type tumours. CRT-analysis established a cut off of high (>0.75, n = 19) and low (≤0.75, n = 147) infiltration. (DOCX) [file pone.0156497.s001.docx]

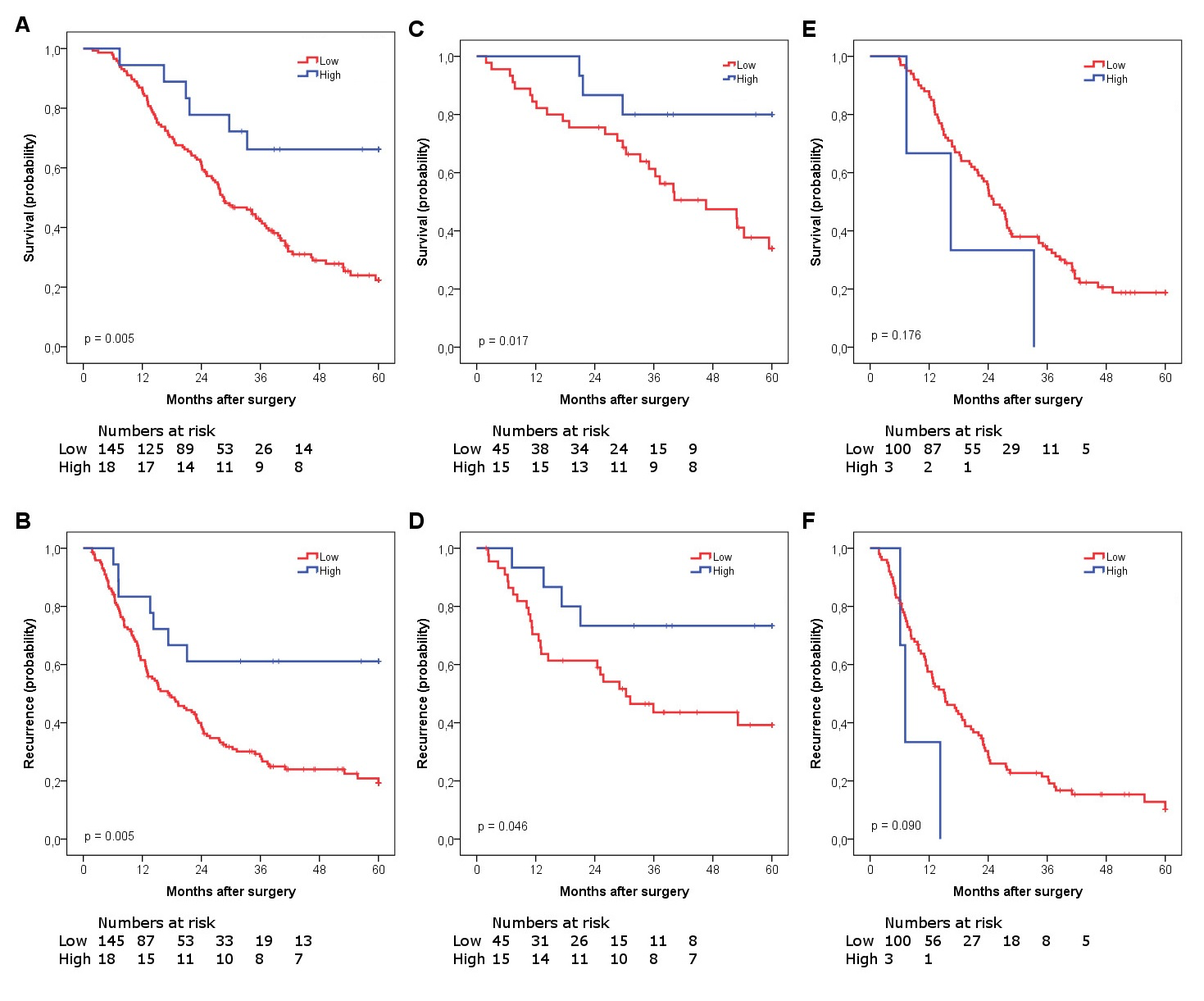
S3 fig: **Kaplan-Meier estimates of survival according to intra-tumour NK-cell density.** Kaplan-Meier estimates of 5-year survival according to intra-tumour CD56+ NK-cell count in (A) the entire cohort, (C) in I-type tumours and(E) in PB-type tumours, and recurrence free survival in (B) the entire cohort, (D) in I-type tumours, and (F) in PB-type tumours. CRT-analysis established a cut off of high (>0.75, n = 19) and low (≤0.75, n = 147) infiltration.
