# Supplementary material for: The Prognostic Impact of NK/NKT Cell Density in Periampullary Adenocarcinoma Differs by Morphological Type and Adjuvant Treatment
Source: PLoS One. 2016 Jun 8;11(6):e0156497. doi: 10.1371/journal.pone.0156497 (PMC4898776; doi:10.1371/journal.pone.0156497)
Supplement: S3 Fig — Kaplan-Meier estimates of 5-year survival according to CD56+ NK/NKT cell/tissue ratio in A) the entire cohort, (C) in I-type tumours and(E) in PB-type tumours, and recurrence free survival in (B) the entire cohort, (D) in I-type tumours, and (F) in PB-type tumours. CRT-analysis established a cut off of high (> 4.722, n = 133) and low (≤4.722, n = 22) infiltration. (DOCX) [file pone.0156497.s003.docx]

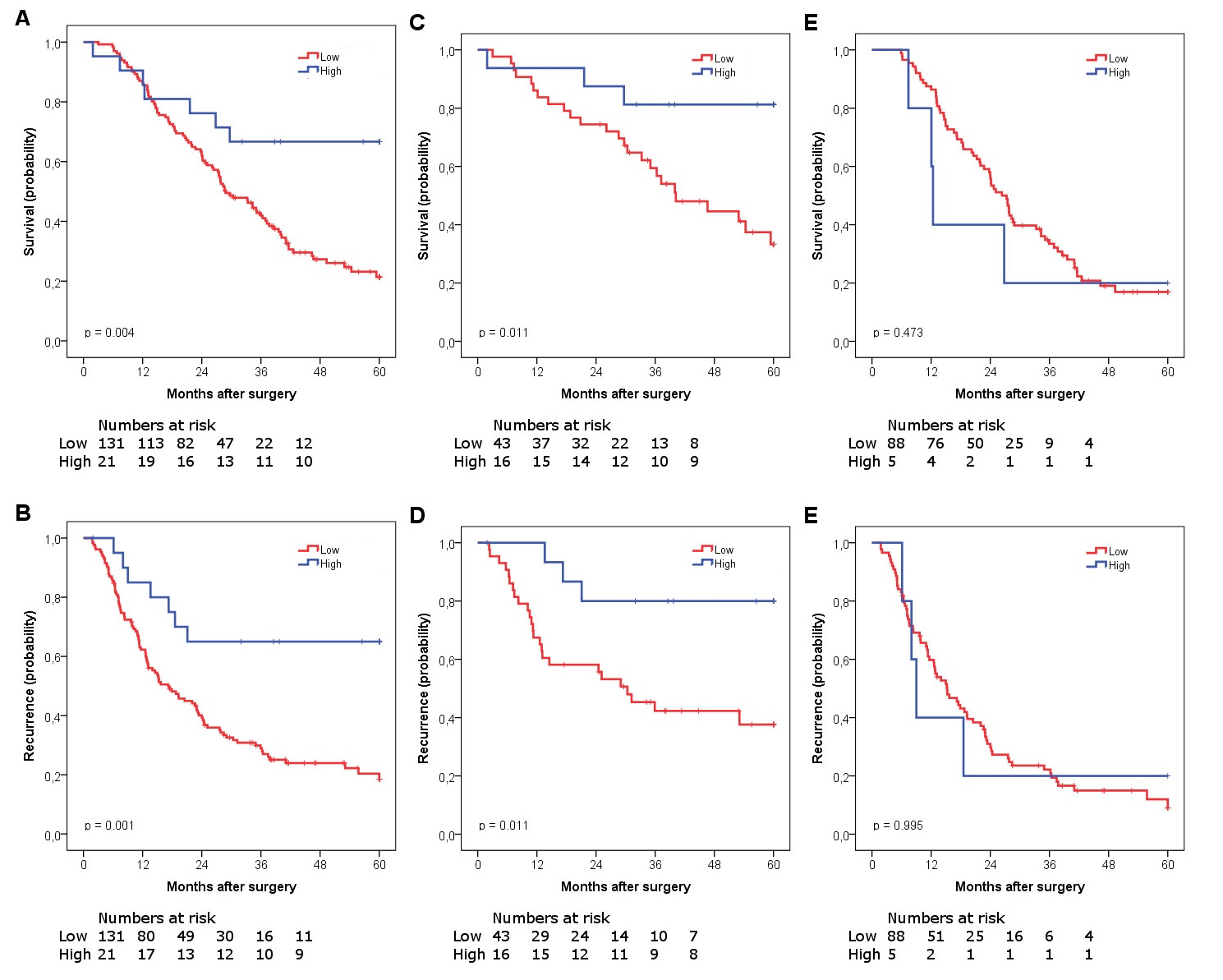
S5 fig: **Kaplan-Meier estimates of survival according to NK-cell /tissue ratio.** Kaplan-Meier estimates of 5-year survival according to CD56+ NK cell/tissue ratio in A) the entire cohort, (C) in I-type tumours and(E) in PB-type tumours, and recurrence free survival in (B) the entire cohort, (D) in I-type tumours, and (F) in PB-type tumours. CRT-analysis established a cut off of high (> 4.722, n = 133) and low (≤4.722, n = 22) infiltration.
