# Supplementary material for: The Prognostic Impact of NK/NKT Cell Density in Periampullary Adenocarcinoma Differs by Morphological Type and Adjuvant Treatment
Source: PLoS One. 2016 Jun 8;11(6):e0156497. doi: 10.1371/journal.pone.0156497 (PMC4898776; doi:10.1371/journal.pone.0156497)
Supplement: S4 Fig — Kaplan-Meier estimates of 5-year survival according to tumour-specific CD56 expression in A) the entire cohort, (C) in I-type tumours and(E) in PB-type tumours, and recurrence free survival in (B) the entire cohort, (D) in I-type tumours, and (F) in PB-type tumours). (DOCX) [file pone.0156497.s004.docx]

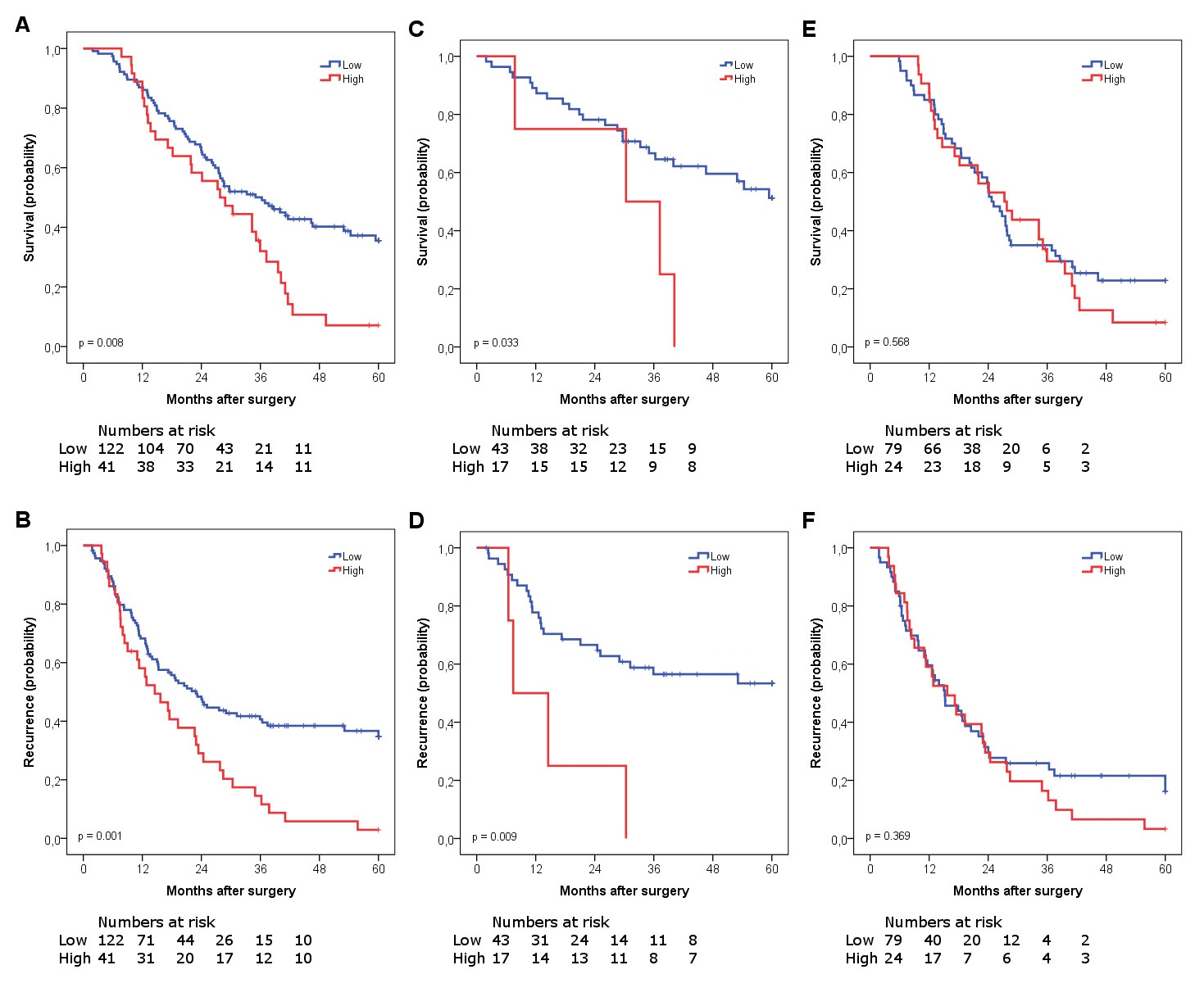
S6 Fig: **Kaplan-Meier estimates of survival according to tumour-specific CD56 expression.**  Kaplan-Meier estimates of 5-year survival according to tumour-specific CD56 expression in A) the entire cohort, (C) in I-type tumours and(E) in PB-type tumours, and recurrence free survival in (B) the entire cohort, (D) in I-type tumours, and (F) in PB-type tumours).
