# Supplementary figures and images for: The Prognostic Impact of NK/NKT Cell Density in Periampullary Adenocarcinoma Differs by Morphological Type and Adjuvant Treatment
Source: PLoS One. 2016 Jun 8;11(6):e0156497. doi: 10.1371/journal.pone.0156497 (PMC4898776; doi:10.1371/journal.pone.0156497)

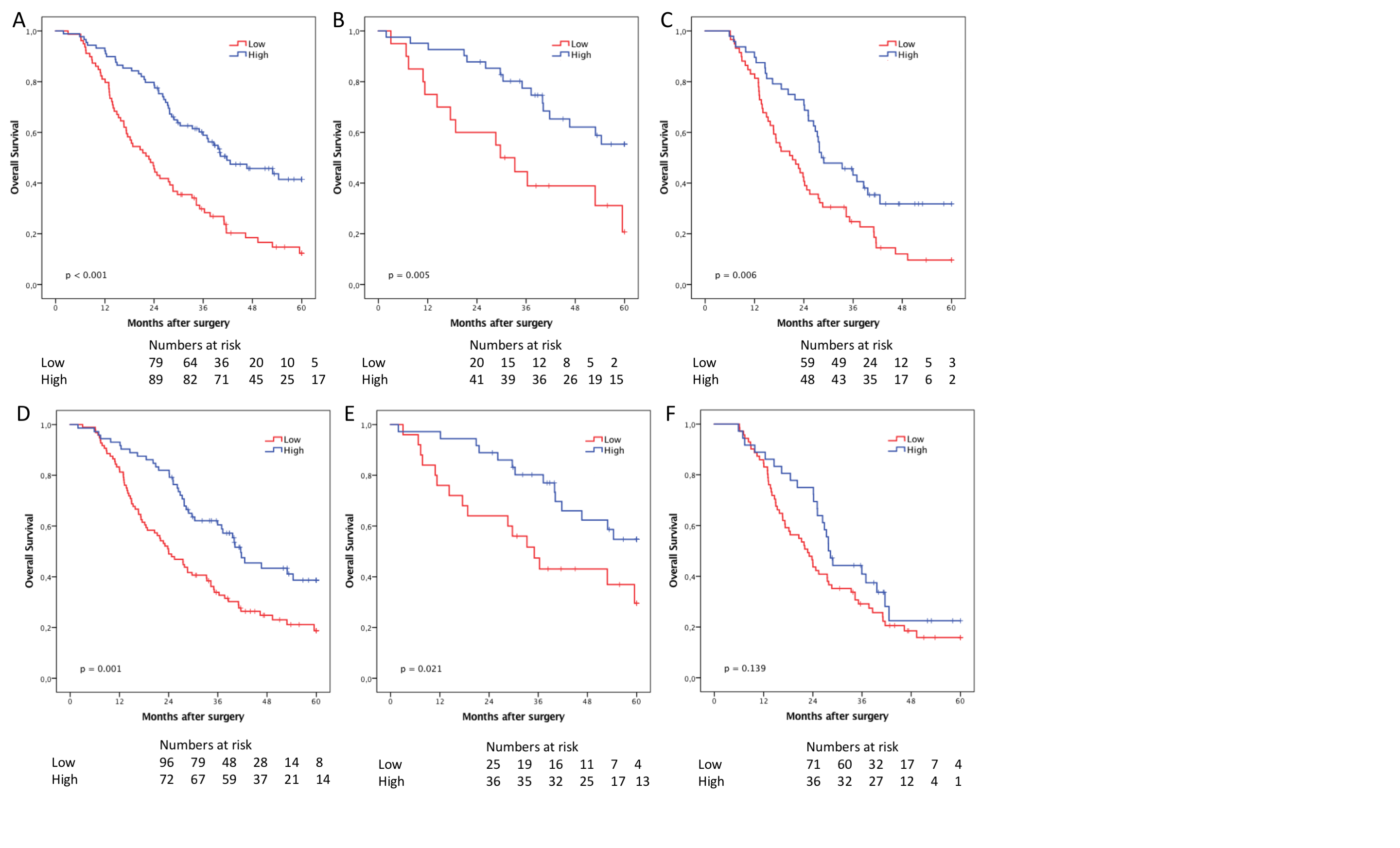

Supplement: S5 Fig — Kaplan-Meier estimates of 5-year survival according to CD3+ lymphocyte infiltration in A) the entire cohort, (B) in I-type tumours, (C) in PB-type tumours, (D) automated analysis of CD3+ infiltration in whole cohort, (E) automated analysis of CD3+ infiltration in I-type and (F) automated analysis of CD3+ infiltration in PB-type. (TIFF) [file pone.0156497.s005.tiff]

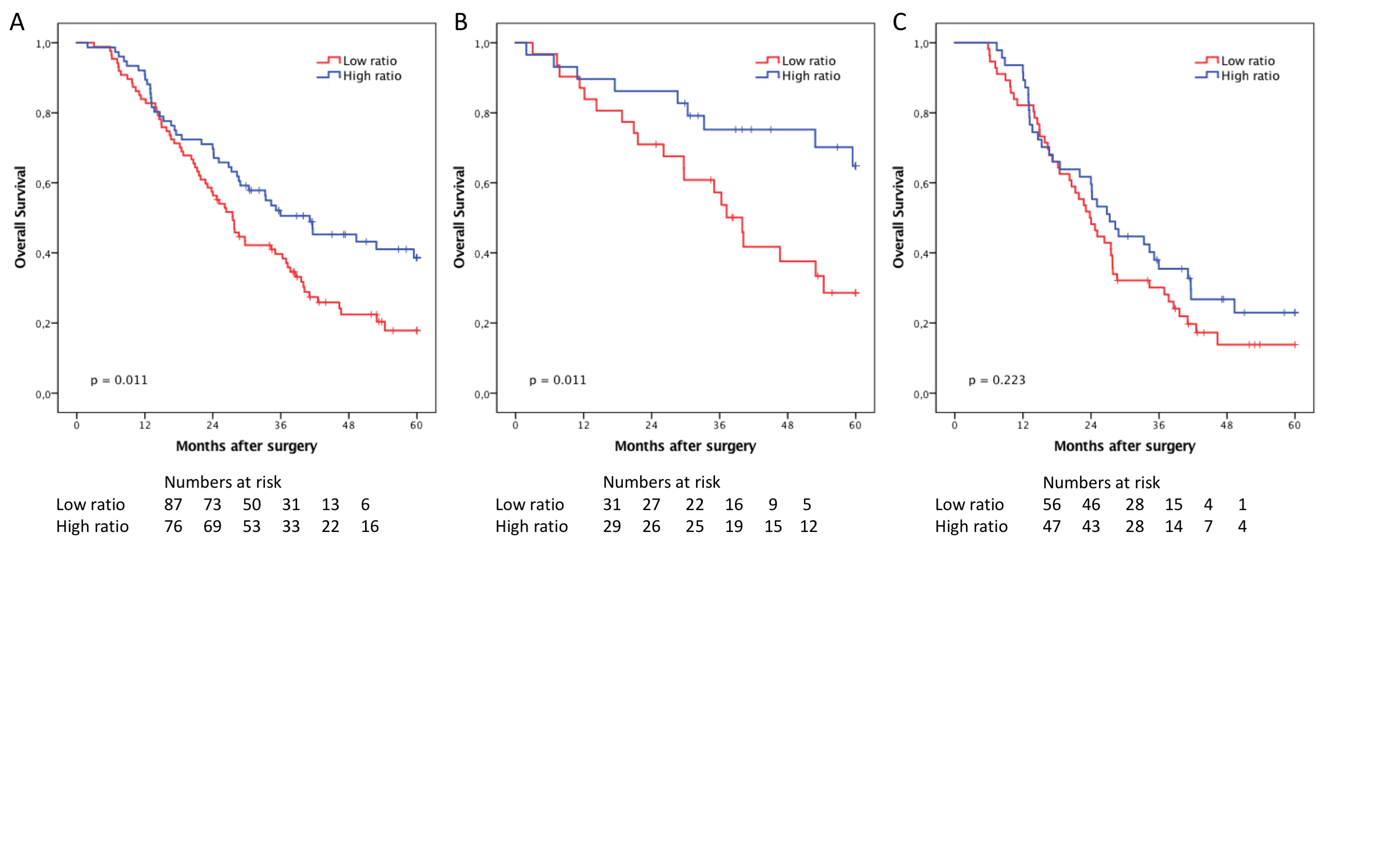

Supplement: S6 Fig — Kaplan-Meier estimates of 5-year survival according to CD56+ lymphocyte to CD3+ lymphocyte infiltration ratio in A) the entire cohort, (B) in I-type tumours and (C) in PB-type tumours. (TIFF) [file pone.0156497.s006.tiff]

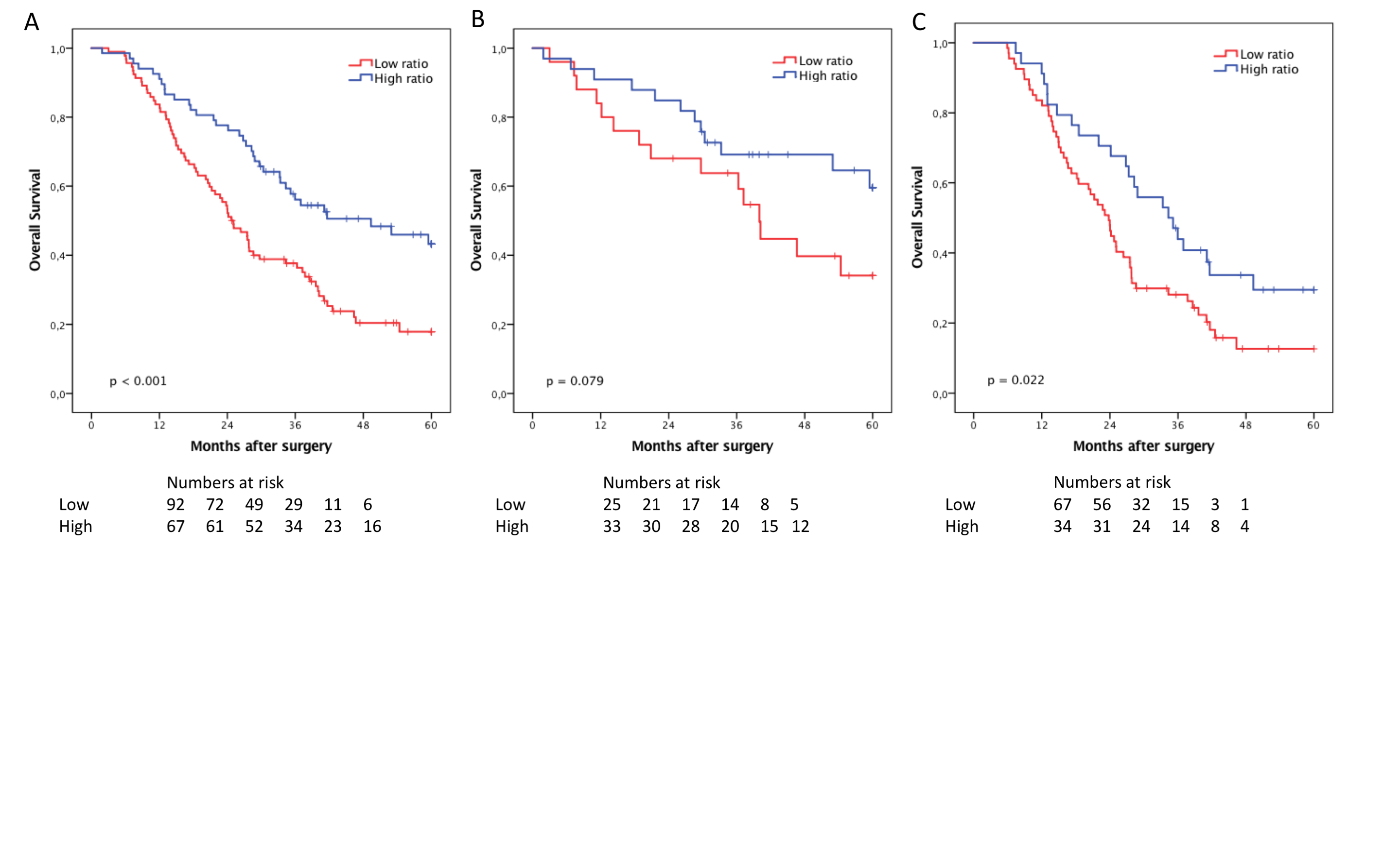

Supplement: S7 Fig — Kaplan-Meier estimates of 5-year survival according to CD56+ lymphocyte to CD1a+ and CD68+ immune cell infiltration in A) the entire cohort, (B) in I-type tumours and (C) in PB-type tumours. (TIFF) [file pone.0156497.s007.tiff]

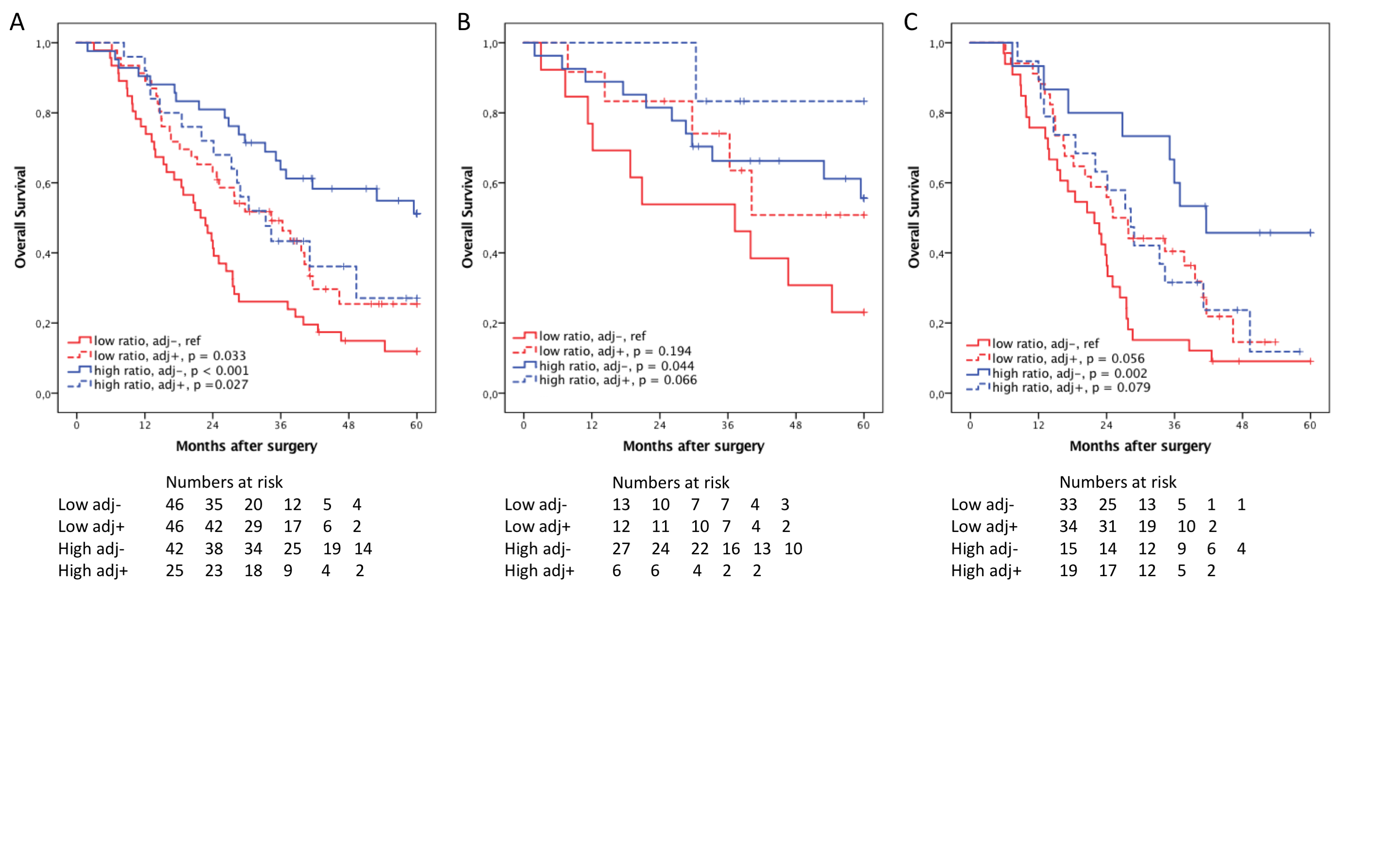

Supplement: S8 Fig — Kaplan–Meier estimates of 5-year survival in combined strata according to CD56+ lymphocyte to CD1a+ and CD68+ immune cell infiltration ratio and adjuvant chemotherapy in (A) the whole cohort, (B) in I-type tumours and (C) in PB-type tumours. (TIFF) [file pone.0156497.s008.tiff]
