# Supplementary material for: The Prognostic Impact of NK/NKT Cell Density in Periampullary Adenocarcinoma Differs by Morphological Type and Adjuvant Treatment
Source: PLoS One. 2016 Jun 8;11(6):e0156497. doi: 10.1371/journal.pone.0156497 (PMC4898776; doi:10.1371/journal.pone.0156497)
Supplement: S1 Table — (DOCX) [file pone.0156497.s009.docx]

S1 Table: Associations between CD56+ tumour tissue fraction and clinicopathological factors.

| Factor | Total CD56 median (range) | p-value |
| --- | --- | --- |
| Age* |  | 0.0334 |
| Q1 (n = 36) | 0.00 (0.00-50.00) |  |
| Q2 (n = 38) | 0.00 (0.00-100.00) |  |
| Q3 (n = 41) | 0.00 (0.00-90.00) |  |
| Q4 (n = 37) | 0.00 (0.00-100.00) |  |
| Sex |  | 0.639 |
| Female (n = 78) | 0.00 (0.00-90.00) |  |
| Male (n = 77) | 0.00 (0.00-100.00) |  |
| Tumour origin |  | **0.001** |
| Duodenum (n = 13) | 0.00 (0.00-100.00) |  |
| Papilla-ampulla intestinal type (n = 48) | 0.00 (0.00-20.00) |  |
| Papilla-ampulla pancreatobiliary type (n = 15) | 0.00 (0.00-100.00) |  |
| Distal bile duct (n = 40) | 0.00 (0.00-90.00) |  |
| Pancreas (n = 39) | 3.50 (0.00-100.00) |  |
| Differentiation grade |  | 0.426 |
| Poor (n = 65) | 0.00 (0.00-100.00) |  |
| Well and moderate (n = 90) | 0.00 (0.00-100.00) |  |
| Tumour stage |  | 0.804 |
| T1 and T2 (n = 25) | 0.00 (0.00-90.00) |  |
| T3 and T4 (n = 130) | 0.00 (0.00-100.00) |  |
| Nodal stage |  | 0.182 |
| N1 (n = 58) | 0.00 (0.00-100.00 |  |
| N2 (n = 58) | 0.00 (0.00-100.00 |  |
| N2 (n = 39) | 0.00 (0.00-100.00) |  |
| Resection margins |  | **0.046** |
| R0 (n = 22) | 0.00 (0.00-50.00) |  |
| R1 (n = 84) | 0.00 (0.00-100.00) |  |
| RX (n = 49) | 0.00 (0.00-100.00) |  |
| Perineural growth |  | 0.128 |
| Absent (n = 63) | 0.00 (0.00-100.00) |  |
| Present (n = 92) | 0.00 (0.00-100.00) |  |
| Lymphatic growth |  | 0.593 |
| Absent (n = 57) | 0.00 (0.00-100.00) |  |
| Present (n = 98) | 0.00 (0.00-100.00) |  |
| Vascular growth |  | 0.739 |
| Absent (n = 116) | 0.00 (0..00-100.00) |  |
| Present (n = 39) | 0.00 (0.00-100.00) |  |
| Peripancreatic fat growth |  | **0.006** |
| Absent (n = 59) | 0.00 (0.00-100.00 |  |
| Present (n = 96) | 0.00 (0.00-100.00) |  |

* Q1 = 38-61, Q2 = 62-67, Q3 = 68-72, Q4 = 73-84
