# Supplementary material for: The Prognostic Impact of NK/NKT Cell Density in Periampullary Adenocarcinoma Differs by Morphological Type and Adjuvant Treatment
Source: PLoS One. 2016 Jun 8;11(6):e0156497. doi: 10.1371/journal.pone.0156497 (PMC4898776; doi:10.1371/journal.pone.0156497)
Supplement: S2 Table — (DOCX) [file pone.0156497.s010.docx]

S2 Table: Cox proportional hazards analysis of the impact of intra-tumoural CD56+ NK-cells, stromal NK-cells, NK-cell/tissue ratio and tumour-specific CD56 expression, respectively, on overall survival and recurrence-free survival.

|  | Overall survival | | Recurrence-free survival | |
| --- | --- | --- | --- | --- |
|  | HR (95% CI) | | HR (95% CI) | |
|  | Unadjusted | Adjusted | Unadjusted | Adjusted |
| Intra-tumoural NK-cell count, entire cohort | **0.33 (0.14-0.75)** | 0.65 (0.26-1.61) | **0.36 (0.17-0.78)** | 0.79 (0.36-1.87) |
| Intra-tumoural NK-cell count, I-type | **0.26 (0.08-0.87)** | 0.35 (0.08-1.47) | 0.36 (0.13-1.05) | 0.93 (0.21-4.05) |
| Intra-tumoural NK-count, PB-type | 2.25 (0.70-7.18) | 3.11 (0.86-11.17) | 2.80 (0.87-9.02) | 3.11 (0.86-11-17) |
| Stromal NK-count, entire cohort | **0.61 (0.39-0.98)** | **0.58 (0.34-0.97)** | 0.65 (0.42-1.02) | 0.82 (0.50-1.34) |
| Stromal NK-count, I-type | 0.95 (0.32-2.83) | 0.11 (0.10-1.14) | 0.80 (0.23-2.74) | 1.03 (0.11-9.86) |
| Stromal NK-count, PB-type | 0.62 (0.37-1.06) | 0.57 (0.31-1.05) | 0.75 (0.46-1.23) | 0.80 (0.45-1.39) |
| NK-cell ratio, entire cohort | **0.39 (0.23-0.64)** | 0.61 (0.35-1.08) | **0.30 (0.18-0.50)** | **0.56 (0.31-0.99)** |
| NK-cell ratio, I-type | 0.95 (0.32-2.83) | 0.33 (0.10-1.12) | 0.80 (0.23-2.74) | 0.46 (0.13-1.63) |
| NK-cell ratio, PB-type | 0.62 (0.37-1.06) | 0.80 (0.42-1.55) | 0.75 (0.46-1.23) | 0.76 (0.40-1.45) |
| Tumour-specific CD56 expression, entire cohort | **1.77 (1.16-2.72)** | **1.60 (1.03-2.50)** | **1.99 (1.31-3.00)** | **1.83 (1.19-2.81)** |
| Tumour-specific CD56 expression, I-type | **3.05 (1.04-8.94)** | 0.77 (0.17-3.66) | **3.78 (1.29-11.12)** | 1.32 (0.26-6.60) |
| Tumour CD56 expression, PB-type | 1.15 (0.71-1.86) | 1.35 (0.81-2.25) | 1.24 (0.78-1.96) | 1.51 (0.93-2.46) |

Adjusted analysis included age (continuous), T-stage (1-2 vs 3-4), N-stage, differentiation grade (poor vs well-moderate), lymphatic invasion, vascular invasion, perineural growth and adjuvant therapy (yes vs no). Bold text indicates significant values.
